# Validation of a pulmonary embolism risk assessment model in gynecological inpatients: Clinical trial: A single-center, retrospective study

**DOI:** 10.1186/s12959-024-00616-5

**Published:** 2024-06-05

**Authors:** Zhen-Yi Jin, Chun-Min Li, Hong Qu, Wen-Tao Yang, Jia-Hao Wen, Hua-Liang Ren

**Affiliations:** Department of Vascular Surgery, Beijing Chaoyang Hospital, Capital Medical University, No.8 Gongti South Road, Beijing, 100020 China

**Keywords:** Caprini, Gynecological, Inpatient, Pulmonary embolism (PE), PADUA

## Abstract

**Objective:**

To compare the predictive efficacy of the PADUA and Caprini models for pulmonary embolism (PE) in gynecological inpatients, analyze the risk factors for PE, and validate whether both models can effectively predict mortality rates.

**Methods:**

A total of 355 gynecological inpatients who underwent computed tomography pulmonary angiography (CTPA) were included in the retrospective analysis. The comparative assessment of the predictive capabilities for PE between the PADUA and Caprini was carried out using receiver operating characteristic (ROC) curves. Logistic regression analysis was used to identify risk factors associated with PE. Additionally, Kaplan–Meier survival analysis plots were generated to validate the predictive efficacy for mortality rates.

**Results:**

Among 355 patients, the PADUA and Caprini demonstrated the area under the curve (AUC) values of 0.757 and 0.756, respectively. There was no statistically significant difference in the AUC between the two models (*P* = 0.9542). Multivariate logistic analysis revealed immobility (*P* < 0.001), history of venous thromboembolism (VTE) (*P* = 0.002), thrombophilia (*P* < 0.001), hormonal treatment (*P* = 0.022), and obesity (*P* = 0.019) as independent risk factors for PE. Kaplan–Meier survival analysis demonstrated the reliable predictive efficacy of both the Caprini (*P* = 0.00051) and PADUA (*P* = 0.00031) for mortality. ROC for the three- and six-month follow-ups suggested that the Caprini model exhibited superior predictive efficacy for mortality.

**Conclusions:**

The PADUA model can serve as a simple and effective tool for stratifying high-risk gynecological inpatients before undergoing CTPA. The Caprini model demonstrated superior predictive efficacy for mortality rates.

**Supplementary Information:**

The online version contains supplementary material available at 10.1186/s12959-024-00616-5.

## Introduction

Pulmonary embolism (PE) and deep vein thrombosis (DVT) represent two clinical manifestations of venous thromboembolism (VTE) [1]. The annual PE incidence is estimated to be 23 cases per 100,000 individuals [2]. PE is the third most common cause of cardiovascular mortality, following myocardial infarction and stroke [3]. Alarmingly, reports indicate that the mortality rate associated with PE in hospitalized patients can reach 71% [4].

Existing research highlights a heightened incidence of PE in females compared to males [5–8]. This gender discrepancy may be attributed to anatomical and hormonal factors, although further investigation is needed [9, 10]. Among patients diagnosed with PE, women were more likely to have severe clinical features such as hypotension and more frequent evidence of right ventricular dysfunction on echocardiography [11]. In addition, thrombus management is also important for women. Although anticoagulation is effective in treating PE and reducing recurrence in both men and women, women receiving anticoagulation therapy are more likely to experience bleeding complications [5]. Consequently, accurate PE diagnosis and management in gynecological patients is of paramount importance.

Currently, computed tomography pulmonary angiography (CTPA) is considered the gold standard for diagnosing PE [12]. However, this diagnostic procedure has the drawbacks of exposing patients to radiation, incurring substantial costs, and posing risks, such as contrast-induced nephropathy [13]. There could be a sex-based difference in the efficacy of diagnosis of PE using a CTPA [10]. Previous research has indicated women who are ultimately tested for PE using CTPA are 35%–55% less likely to be diagnosed with PE compared to similarly aged men [11]. Therefore, considering the specificity of the gynecological population, there is a pressing need for the development of a risk assessment model (RAM) specifically tailored for gynecological inpatients, aiming to screen for PE and thereby minimize the unnecessary utilization of CTPA.

The PADUA and Caprini RAMs have been extensively examined in academic literature [14, 15]. PADUA is one of the recognized scoring methods for assessing the risk of VTE during hospitalization for non-surgical patients [16]. PADUA is based on 11 criteria, each with its own score, and the cumulative score determines the risk level. In the PADUA model, individuals with a cumulative score of ≥ 4 are considered to be at a high risk for VTE, while those with a score of < 4 are classified as low-risk [14]. High risk Patients of PADUA model are recommended to receive prophylactic measures primarily involving anticoagulant medications [17]. The PADUA model was derived from a cohort study conducted in Italy, and some scholars have expressed concerns regarding the representativeness of the patients included in this study [18]. However, existing prospective studies have validated the clinical utility of the PADUA score [19, 20]. The Caprini model categorizes patients into different risk level groups, such as "extremely low risk" (0 points), "low risk" (1–2 points), "moderate risk" (3–4 points), and "high risk" (≥ 5 points) [21]. Evidence has shown the effectiveness of the Caprini model in surgical patients [22, 23]. However, the optimal threshold of Caprini is different according to different clinical departments [24]. The ACCP guidelines recommend adopting a Caprini for gynecological patients [21].Whereas, the majority of gynecologic patients in the Caprini were at moderate or high risk [25], the applicability of Caprini in gynecologic surgical patients remains controversial [26].

Accurately identifying high-risk patients for PE is crucial for disease management and reducing unnecessary CTPA procedures. Given the specific characteristics of the patient population, selecting a RAM for predicting PE is necessary for gynecological inpatients. The present study assessed and compared the effectiveness of the PADUA and Caprini models in stratifying the risk of PE among gynecological inpatients, as well as their performance in predicting mortality.

## Methods

### Study design and patients

Medical records of gynecological inpatients who underwent CTPA between December 2018 and December 2022 at Beijing Chao-Yang Hospital were selected for the present study. Release of information from this database did not require the patients’ explicit consent. The study received approval from the Research Ethics Committee of Beijing Chaoyang Hospital, Capital Medical University (2023-ke-12).

**Fig. 1:**
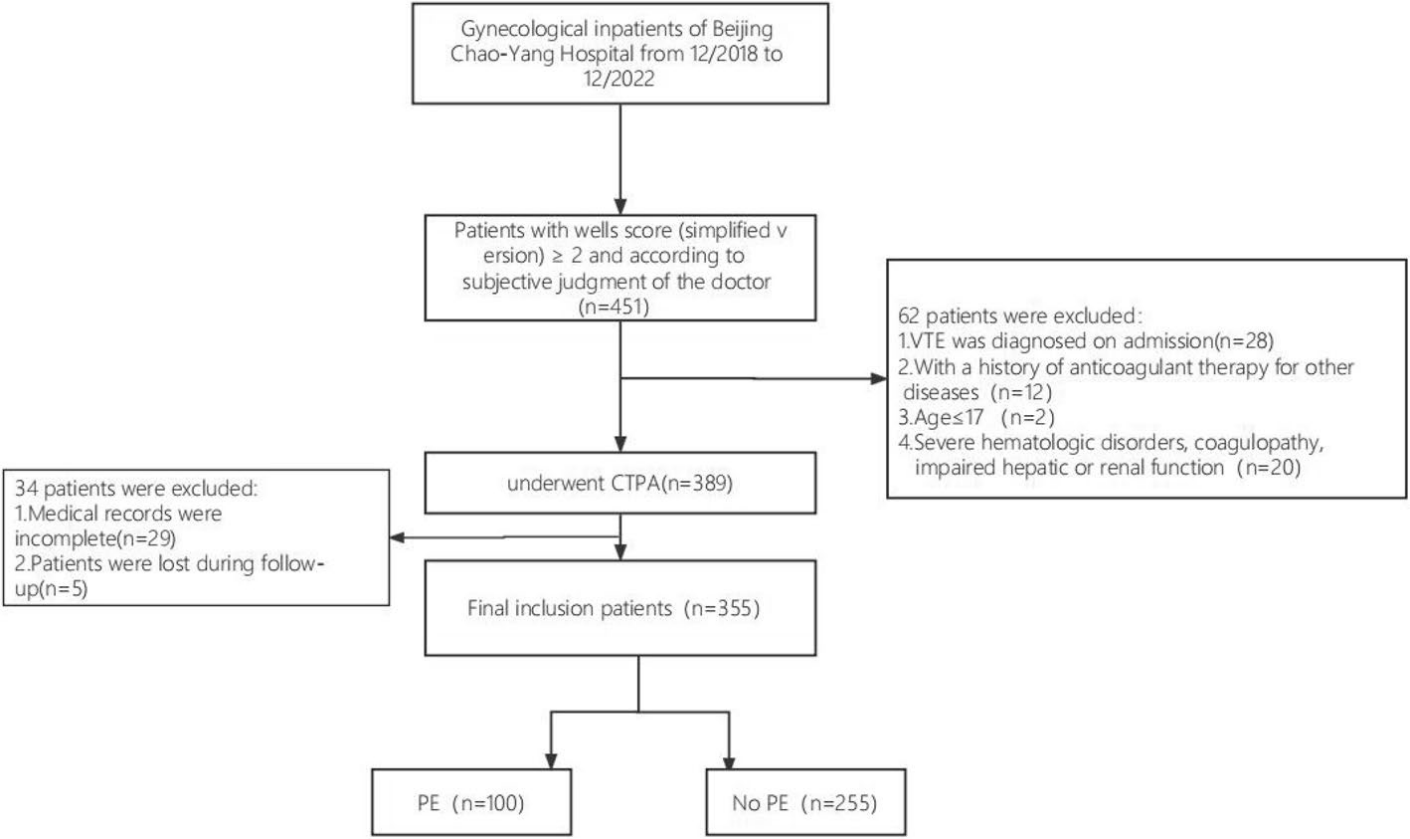
Flow chart

The following inclusion criteria were used in the course of the study: 1) gynecological inpatients; 2) patients with a Wells score (simplified version) of ≥ 2 with suspicion of PE based on the subjective assessment of the physician; 3) age of ≥ 18 years; and 4) hospitalization duration of ≥ 2 days. The exclusion criteria were: 1) diagnosed VTE upon admission; 2) patients receiving anticoagulant therapy before admission for other conditions, such as cardiovascular diseases; 3) age of < 18 years; 4) severe hematologic disorders, coagulation dysfunction, and compromised and/or damaged liver and kidney function; and 5) incomplete medical records. A total of 355 gynecological inpatients were included in the final analysis based on these criteria (Fig. 1).

### Data collection

Clinical data were systematically collected by trained medical staff using standardized case report forms to ensure data reliability. Demographic characteristics, medical histories, and data for occurrences of PE and factors required for calculating the PADUA and Caprini models were collected for all patients. A retrospective evaluation of PE risk was carried out for each patient included in the study utilizing the PADUA and Caprini RAMs based on the available patient information. Furthermore, six-month follow-up was conducted for all included patients (with the first day of hospitalization considered as day 1), during which data on all-cause mortality were collected. Follow-ups were conducted at 1, 3, and 6 months as needed in conjunction with outpatient visits and hospital treatments. Causes of death were determined based on death certificates and autopsy reports.

### Diagnostic criteria

DVT was diagnosed based on Doppler ultrasound and/or venography, while PE was diagnosed based on the CTPA examination. PE was considered to be central if it was located in the main, mediastinal, or lobar pulmonary arteries, or peripheral if the embolus was only in the segmental or subsegmental arteries [27]. Lower extremity vascular ultrasound covered the region from the distal iliac vein to the calf veins. One or more DVTs in the distal popliteal vein were defined as isolated distal DVT (IDDVT) [28]. Proximal DVT (PDVT) was defined as thrombosis in the popliteal, femoral, iliac, and/or inferior vena cava veins. Ultrasound confirmed acute thrombosis if there was no blood flow in the non-compressible venous segment or lumen or if echogenic material was present [29].

### Statistical analyses

Assessment of continuous variable normality was conducted using the Shapiro–Wilk test. Normally distributed data were compared using independent sample t-tests and results were described using means ± standard deviations. Non-normally distributed data were analyzed using the Mann–Whitney U test and results were presented as medians (P25–P75). Categorical data were represented as numbers (%) and comparisons were made using the χ2 test. Receiver operating characteristic (ROC) curves were plotted for both models to predict PE and the corresponding area under the curve (AUC) was calculated. The optimal cutoff values for predicting PE using PADUA and Caprini model were obtained by the Youden Index of ROC curve, then the sensitivity and specificity value were obtained when this cutoff value is taken. Diagnostic test statistics were then computed and included positive predictive value (PPV), negative predictive value (NPV), and overall accuracy. Logistic regression analysis was employed to explore the risk factors for PE. Kaplan–Meier curves were used to determine the time course of death events in the study patients based on the PADUA and Caprini RAMs, with group comparisons performed using the log-rank test. All statistical analyses were conducted using SPSS Statistics v.19 software (IBM, Armonk, NY, USA).

## Results

### Baseline characteristics of the study population

A total of 355 gynecological inpatients who underwent CTPA examinations participated in the study. Their demographic characteristics are summarized in Table 1. Patients in the included population were categorized into the PE and no-PE groups, with 100 cases (28.2%) of diagnosed PE. There were no statistically significant differences in age between the two groups. The PE group had a higher proportion of obese patients (25.0% vs. 13.7%, *P* = 0.012). The PADUA (3.0 [2-4] vs. 2.0 [1-3], *P* < 0.001) and Caprini (7.0 [6-9] vs. 5.0 [4-6], *P* < 0.001) model scores were both higher in the PE group compared to those in the no-PE group. The proportions of patients with active cancer, history of VTE, and thrombophilia in the PE group were significantly higher than no-PE group(60.0% vs. 45.1%, *P* = 0.012; 19.0% vs. 6.7%, *P* < 0.001; 28.0% vs. 1.6%, *P* < 0.001). PE patients were more likely to receive hormonal treatment than no-PE patients (44.0% vs. 24.3%, *P* < 0.001). The immobilization duration was significantly longer for patients with PE compared to those without PE (41.0% vs. 3.9%, *P* < 0.001). There were no statistically significant disparities observed in the number of patients undergoing surgery between the two groups. The PE group also exhibited a lengthier surgery duration (4.1 vs. 3.4, *P* = 0.037) (Table 2).

**Table 1:** Covariates in the PADUA and Caprini Scores

	PADUA	Caprini
**Age**	≥ 70:1	41–60:1 61–74:2 ≥ 75:3
**BMI**	≥ 30:1	BMI ≥ 25:1
**Medical History**		
Cancer	Active cancer: 3	Current or past malignancy: 2
Previous VTE	3	3
Known thrombophilic condition	3	3
Inflammatory bowel disease	NA	1
Visible varicose veins	NA	1
Family history of VTE	NA	3
Abortion	NA	1
HIT	NA	3
**Recent condition**		
Surgery	< 30 d:2	Major surgery(< 30d):1 Major surgery(< 1h):2 Major surgery(2-3h):3
Trauma		NA
Reduced mobility	≥ 3d:3	On bed:1 > 72h:2
Heart failure	1	1
Respiratory failure		1
MI	1	1
Stroke	ischemic	5
Acute infection	1	1
Rheumatologic diseases		NA
Sepsis	NA	1
Hormonal treatment	1	1
Spinal cord injury resulting in paralysis	NA	5
Existing lung disease	NA	1
Pregnancy or puerperium(< 1month)	NA	1
Swollen legs	NA	1
Immobilizing plaster cast in past 1month	NA	2
Central venous access	NA	2
Hip, pelvis, or leg fracture	NA	5
**Gynaecological factors**		
Pregnancy or puerperium(< 1month)	NA	1
History of unexplained stillborn infant, recurrent spontaneous abortion, premature birth with toxemia or growth-restricted infant	NA	1

*NA* Not applicable, items are not part of the risk assessment model

Abbreviations: *BMI* Body Mass Index, *VTE* venous thromboembolism, *HIT* Heparin induced thrombocytopenia, *MI* myocardial infarction

**Table 2:** Patient characteristics

	PE(100)	No PE(255)	*P* value
N(%)			
Age, mean(SD)	63(11.0)	61(12.8)	0.221
BMI, mean(SD)	27.1(4.4)	25.2(4.1)	< .001
Surgery, n(%)	84(84.0)	225(88.2)	0.287
Length of surgery, mean(SD)	4.1(2.8)	3.4(2.6)	0.037
Major surgery(< 1h)	18(18.0)	63(24.7)	0.176
Major surgery(2-3h)	13(13.0)	58(22.7)	0.056
Laparoscopic surgery, n(%)	45(45.0)	102(40.0)	0.435
PADUA score, median(IQR)	3(2–4)	2(1–3)	< .001
Caprini score, median(IQR)	7(6–9)	5(4–6)	< .001
Active cancer, n(%)	60(60.0)	115(45.1)	0.012
Previous VTE, n(%)	19(19.0)	17(6.7)	< .001
Limited mobility, n (%)	41(41.0)	10(3.9)	< .001
Known thrombophilic condition, n (%)	28(28.0)	4(1.6)	< .001
Trauma/surgery within 30 days, n (%)	84(84.0)	225(88.2)	0.287
Heart or respiratory failure, n (%)	2(2.0)	6(2.4)	0.840
Acute ischemic stroke or MI, n (%)	1(1.0)	9(3.5)	0.225
Acute infection or rheumatological disorder, n (%)	15(15.0)	28(11.0)	0.298
Obese (BMI ≥ 30), n (%)	25(25.0)	35(13.7)	0.012
Age ≥ 70, n(%)	28(28.0)	68(26.7)	0.799
DVT, n (%)	90(90.0)	164(64.3)	< .001
Hormonal therapy or oral contraceptives, n (%)	50(50.0)	67(26.3)	< .001
Pregnancy or puerperium(< 1month)	2(2.0)	3(1.2)	0.558
Venous valvular insufficiency or varicose veins, n (%)	6(6.0)	12(4.7)	0.618

*PE* Pulmonary embolism, *BMI* Body mass index, *SD* standard deviation, *VTE* Venous thromboembolism, *DVT* Deep venous thrombosis

### RAM

**Fig. 2:**
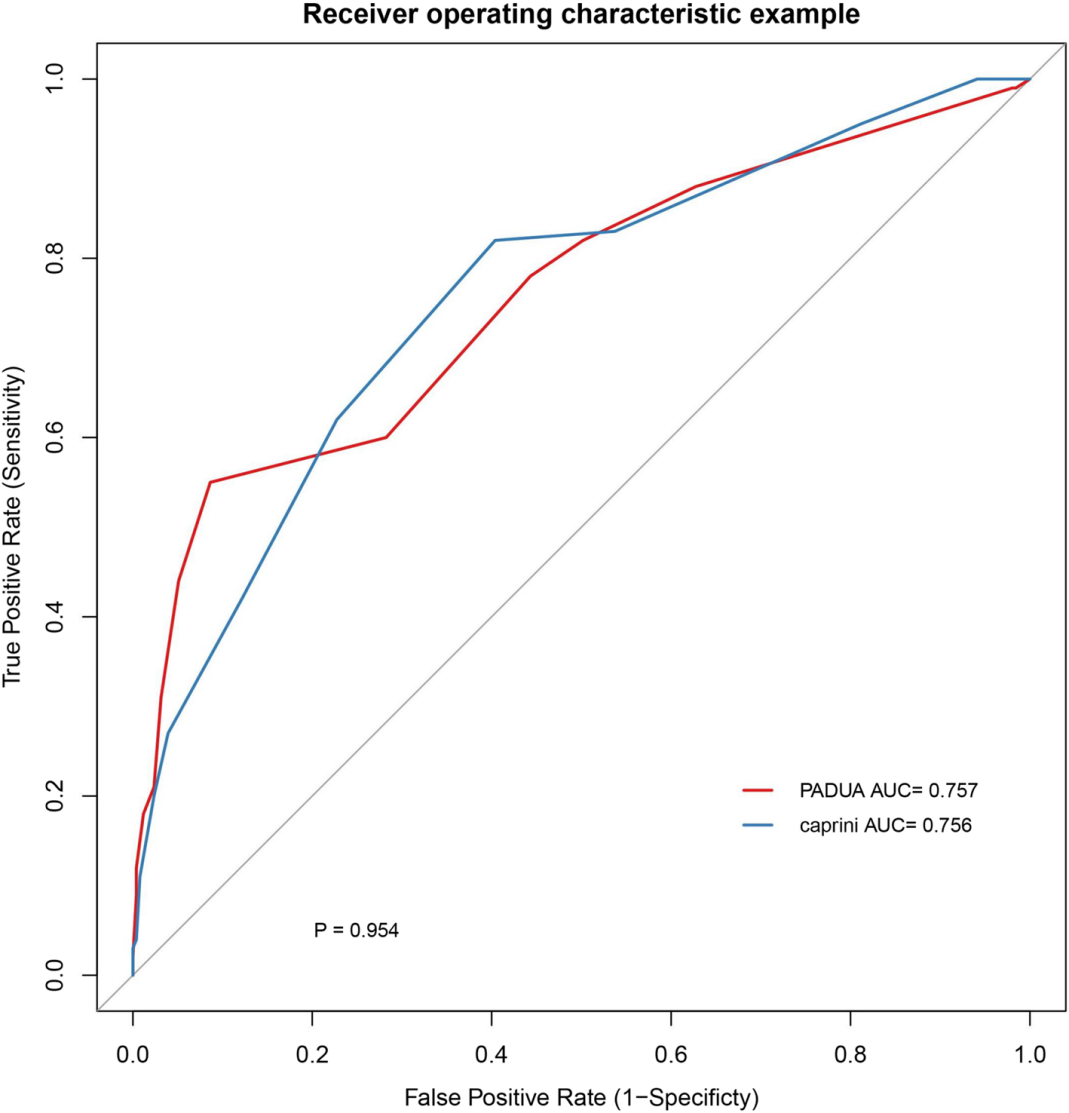
ROC of PADUA and Caprini score. Abbreviations: AUC area under the curve

Figure 2 shows the ROC curves for the PADUA and Caprini model PE prediction. The AUC for the ROC curve for the PADUA model was 0.757 (95% confidence interval (CI) 0.698–0.817), with an optimal cutoff value of 6 or 7. The AUC for the Caprini model was 0.756 (95% CI 0.700–0.813), and the optimal cutoff value was 5 or 6. There was no statistically significant difference in the AUC between the two RAMs (*P* = 0.9542). The summarized indices for both RAMs can be found in Table 3. The PADUA model displayed a sensitivity of 55.0% and specificity of 91.4% in predicting positive PE when using cutoff values of 6 or 7. The PPV was 71.4%, and the NPV was 83.8%. On the other hand, the Caprini model showed a sensitivity of 82.0% and specificity of 59.6% when employing cutoff values of 5 or 6 in predicting positive PE, with a PPV of 44.3% and NPV of 89.4%. Among the 355 patients included in the study, a total of 254 individuals were diagnosed with DVT, with 233 cases classified as IDDVT and 21 cases classified as PDVT. Statistical analysis revealed significant differences in the PADUA (*P* = 0.002) and Caprini (*P* = 0.000) models for both IDDVT and PDVT (Supplemental Table 1). In the subgroup of 100 patients with PE, a statistically significant difference was observed in the PADUA model between central and peripheral PE (*P* = 0.049), while the Caprini model did not show any statistical difference (*P* = 0.271; Supplemental Table 2). In the PE group, the median of PESI score was 1, 26 patients had PE symptoms, 43 patients had hypoxemia, and 14 patients had right heart load. Linear correlation analysis for the two models (PADUA and Caprini) and PESI score showed statistical significance (*P* = 0.000), with Pearson correlation coefficient (R-value) > 0, indicating a positive correlation. The Logistics regression analysis of two models and PE symptoms, hypoxemia and right heart load indicated no statistical significance (Supplemental Table 3).

**Table 3:** Comparison of Padua and Caprini scores

	AUC	Youde n index	Sensitivit y	Specificit y	Positive predictiv e value	Negative predictiv e value	Optimal cutoff
PADU A	0.757 (0.698–0.817)	0.464	0.550	0.914	0.714	0.838	6 OR 7
Caprini	0.756 (0.700–0.813)	0.416	0.820	0.596	0.443	0.894	5 OR 6

*AUC* Area under the curve

### Logistic regression analysis

Logistic regression analysis was employed to examine the risk factors for PE (Table 4). The risk factors were evaluated through both univariate and multivariate analyses. Univariate analysis revealed statistical significance for active cancer (*P* = 0.012), history of VTE (*P* < 0.001), immobilization (*P* < 0.001), thrombophilia (*P* < 0.001), hormonal treatment or oral contraceptives (*P* < 0.001), and obesity (*P* = 0.012). Multivariate analysis showed that, except for active cancer, all other factors maintained statistical significance, indicating that they are independent risk factors for PE in gynecological inpatients.

**Table 4:** Factors associated with pulmonary embolism (PE) in PADUA and Caprini scores

Dependent: PE	OR (univariable)	OR (multivariable)
Age ≥ 70	1.07 (0.64–1.79, *p* = .799)	
Trauma/surgery within 30 days	0.70 (0.36–1.35, * p* = .287)	
Active cancer	**1.83 (1.14–2.92, *p* = .012)**	0.96 (0.38–2.41, *p* = 0.927)
Previous VTE	**3.28 (1.63–6.62, *p* < .001)**	**4.26(1.73–10.49, *p* = 0.002)**
Reduced mobility	**17.03(8.06–35.95, *p* < .001)**	**37.69(15.56–91.29,*p* < .001)**
Known thrombophilic condition	**24.40(8.29–71.85, *p* < .001)**	**50.34(15.05–168.38,*p* < .001)**
Ongoing hormonal treatment or oral contraceptives	**2.81 (1.73–4.54, *p* < .001)**	**2.61 (1.15–5.93, *p* = 0.022)**
Obesity(BMI ≥ 30)	**2.10 (1.18–3.73, *p* = .012)**	**2.59 (1.17–5.76, *p* = 0.019)**
Heart/respiratory failure	0.85 (0.17–4.27, *p* = .840)	
Acute MI or ischemic stroke	0.28 (0.03–2.21, *p* = .225)	
Acute infection/rheum disorder	1.43 (0.73–2.81, *p* = .298)	
Venous valvular insufficiency or varicose veins	1.29 (0.47–3.54, *p* = .618)	
Pregnancy or puerperium(< 1month)	1.71(0.28–10.42, *p *= .558)	

*PE* Pulmonary embolism, *BMI* Body Mass Index, *VTE* venous thromboembolism, *MI* myocardial infarction

### Kaplan–Meier survival analysis

**Fig. 3:**
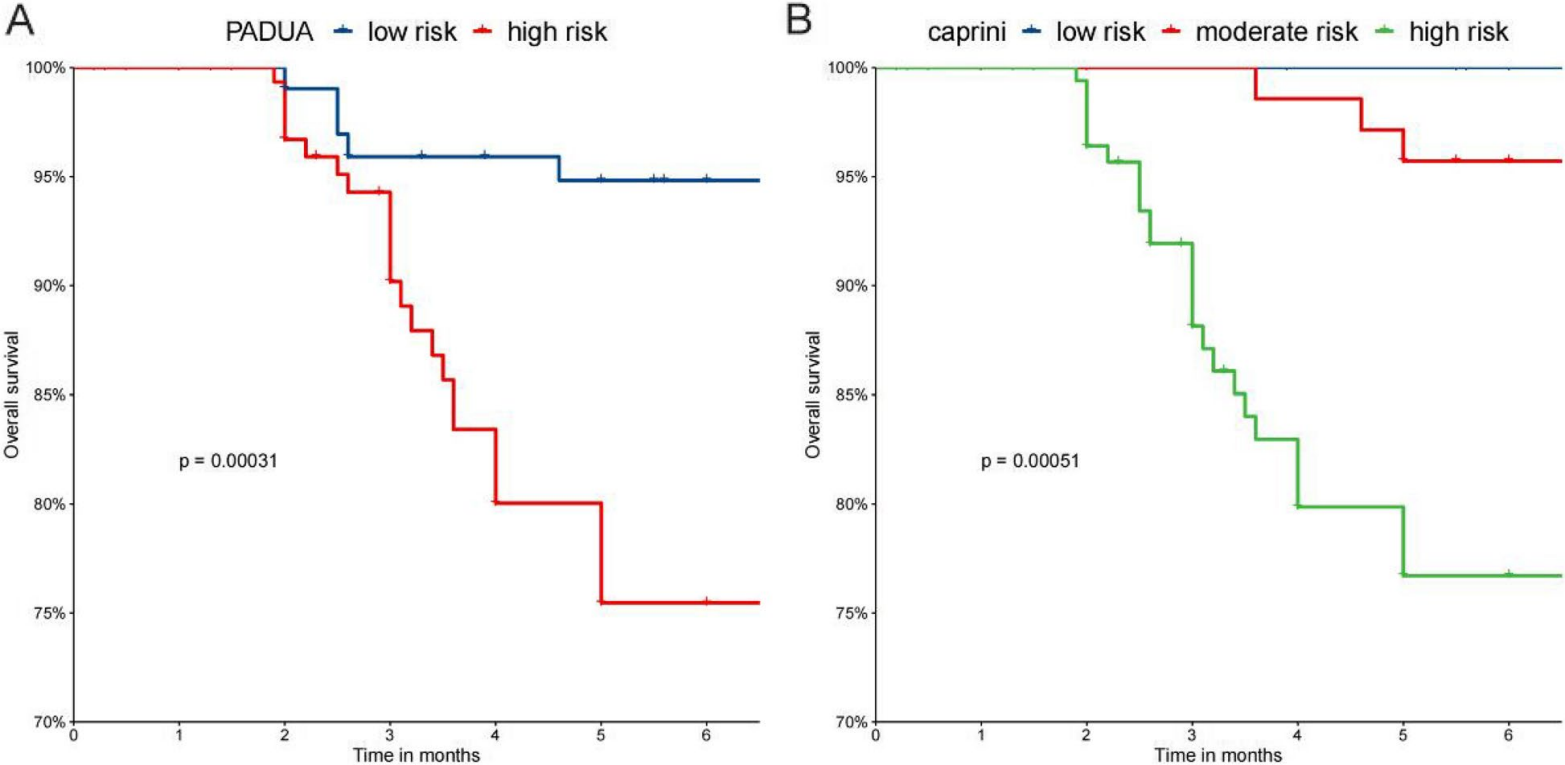
Cumulative survival rates of the patients by PADUA and Caprini risk groups

**Fig. 4:**
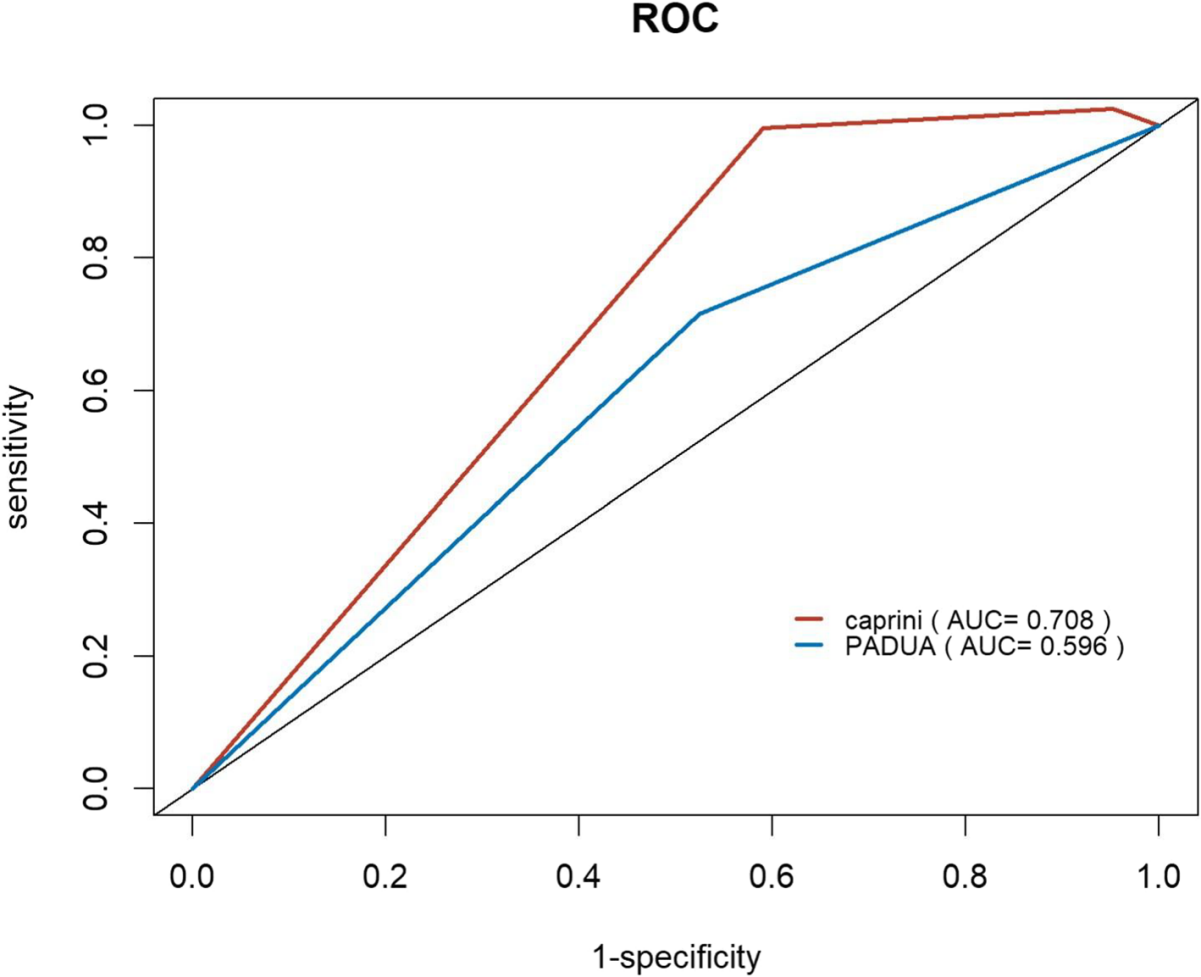
ROC of PADUA and Caprini score in predicting mortality at 3 months follow-up Abbreviations: AUC area under the curve

**Fig. 5:**
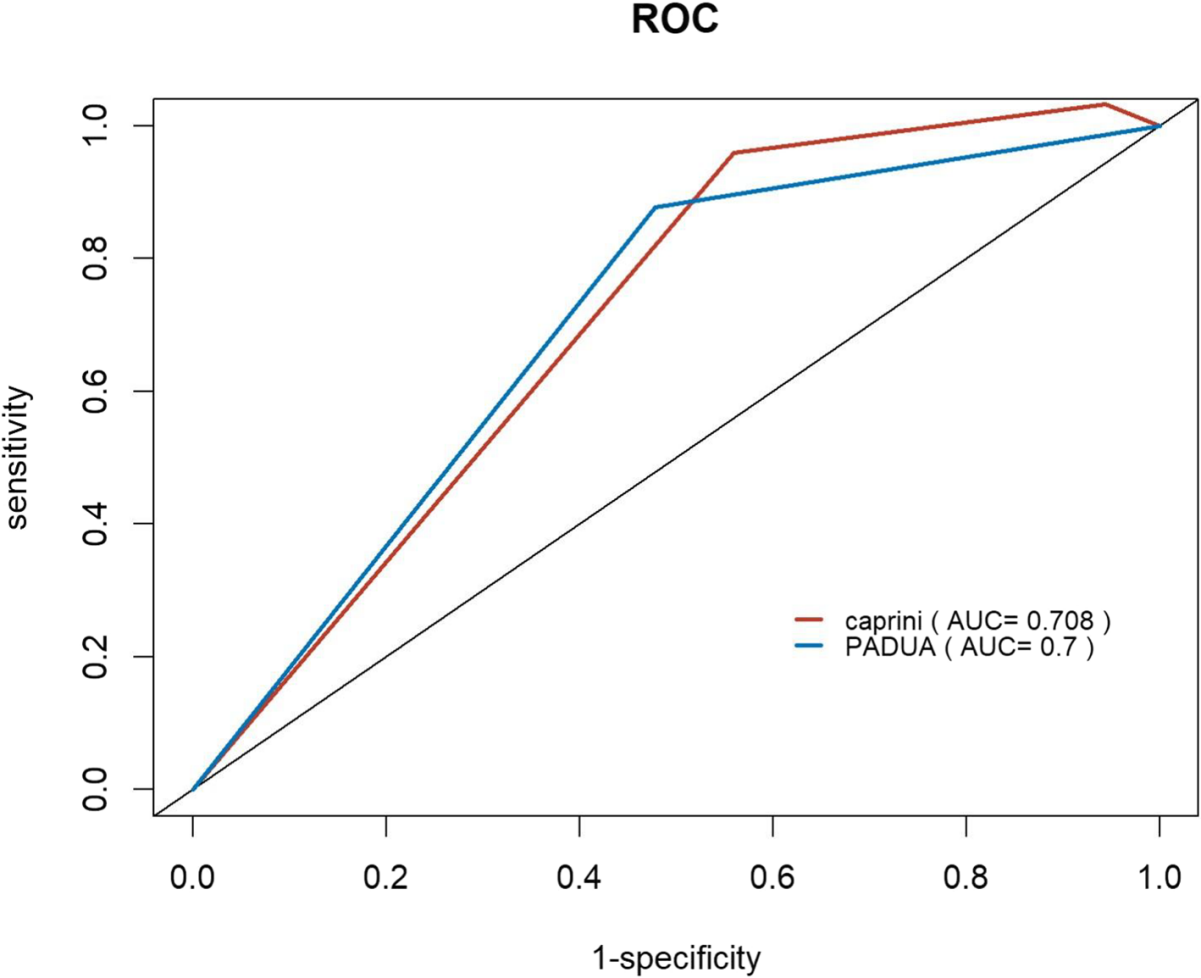
ROC of PADUA and Caprini score in predicting mortality at 6 months follow-up. Abbreviations: AUC area under the curve

Among the 355 included patients, 33 patients (9.3%) died during the six-month follow-up (Table 5). The Kaplan–Meier curves visualize the temporal progression of post-discharge mortality in the included patients based on the PADUA and Caprini RAMs (Figs. 3). Inter-group comparisons were conducted using the log-rank test. The findings from both the PADUA and Caprini RAMs revealed that patients classified as higher-risk exhibited significantly elevated mortality risks compared to those classified as lower-risk. The Caprini model showed significant predictive capability for mortality rates, as evidenced by mortality rates increasing from 0 (0.0%) to 4 (12.1%) and to 29 (87.9%) from low-risk to high-risk (*P* = 0.00031). Similarly, the PADUA model demonstrated mortality rates of 6 (18.2%) and 27 (81.8%) for low- and high-risk patients, respectively (*P* = 0.00051). ROC curves for the PADUA and Caprini RAMs at the three- and six-month follow-ups are shown in Figs. 4 and 5. The AUC for predicting death events at the three-month follow-up was 0.708 for the Caprini model and 0.596 for the PADUA model. At the six-month follow-up, the AUC for predicting death events was 0.708 for the Caprini model and 0.700 for the PADUA model, suggesting that the Caprini model is more effective in predicting death events than the PADUA model.

**Table 5:** Follow-up

	Death	*P* value
Death, n(%)	33	
PADUA low risk(< 4)	6(18.2)	
PADUA high risk(≥ 4)	27(81.8)	0.00051
Caprini low risk(1–2)	0(0.0)	
Caprini moderate risk(3–4)	4(12.1)	
Caprini high risk(≥ 5)	29(87.9)	0.00031

## Discussion

The present study aimed to compare the significance of the Caprini and PADUA models in predicting PE in gynecological inpatients. The results validated the effectiveness and simplicity of the PADUA model as a RAM for predicting PE in this population. Additionally, this study analyzed various risk factors for PE, such as a history of VTE, immobilization, thrombophilia, hormonal treatment or oral contraceptives, and obesity. The Caprini model demonstrated superior predictive efficacy for mortality. Using the PADUA model proved beneficial in the identification of high-risk individuals for PE screening among gynecological inpatients. Conversely, the Caprini model showed exceptional proficiency in predicting mortality, thereby enabling the implementation of timely preventive measures.

### Comparison of two RAMs

Currently, there are several thrombosis RAMs, including the Caprini, PADUA, Khorana, and IMPROVE models, each with distinct areas of focus. The Khorana model is applicable to patients with malignancies [22]. While the Caprini model is primarily designed for assessing thrombosis risk in surgical patients [15], its predictive efficacy is limited when applied to non-surgical and gynecological patients [26, 30]. On the other hand, the PADUA model was validated in a cohort study by Barbar et al. [14]. However, there is limited research specifically validating the PADUA RAM in hospitalized patients [31]. The IMPROVE model predicts the risk of thrombosis formation in acutely hospitalized patients [32]. Gynecological inpatients, who encompass a distinct population comprising both surgical and non-surgical cases, necessitate the development of a specialized prediction model for postoperative PE. Within the scope of this investigation, a total of 309 individuals (87.0%) underwent surgical intervention among the cohort under examination. The PADUA and Caprini models were evaluated and contrasted to formulate an appropriate RAM tailored specifically for gynecological inpatients.

In the present study, both the PADUA and Caprini models demonstrated good predictive efficacy for PE, with AUC values of 0.757 (95% CI 0.698–0.817) and 0.756 (95% CI 0.700–0.813), respectively. The AUC curves of the two models showed no statistically significant disparity (*P* = 0.9542). In contrast to the Caprini model, the PADUA model includes fewer components, featuring a more straightforward age and surgery stratification. The PADUA model incorporates an additional factor of trauma occurring within a 30-day period. Existing research posits that the probability of trauma in females might be lower than in males due to the differences in occupation and environmental exposure [33, 34]. Hence, it can be inferred that the PADUA model demonstrates sufficient efficacy in predicting PE events in gynecological inpatients when compared to the more intricate Caprini model. Central PE poses a grave threat to life, even resulting in mortality [35], and has a significantly worse prognosis than peripheral PE. Barco et al. suggested that the recurrence and mortality risks of PDVT are higher than those of IDDVT [36]. Thus, the site of thrombus formation can be regarded as an indicator of VTE severity. In the present study, the PADUA model showed statistical differences in both groups, while the Caprini model only exhibited statistical differences in IDDVT and PDVT. Consequently, it seems that the PADUA model is more appropriate for predicting VTE severity. On the other hand, simplified PESI (sPESI) score is a practical validated score aimed to stratify 30-day mortality risk in acute PE [37]. The results of this study suggest that PADUA and Caprini models are linearly correlated with sPESI (*P* = 0.000,R > 0), indicating that these two models also can predict the prognosis of PE. Barbar et al. defined a score of ≥ 4 in the PADUA model as high risk for VTE [14]. In the present study, the optimal cutoff values for predicting PE in gynecological inpatients were 6 or 7. The study population encompassed several risk factors associated with VTE, such as surgical procedures (87.0%), active malignancy (49.3%), obesity (16.9%), and various other factors. The observed increase in the optimal cutoff values can be attributed to the enrollment of patients deemed to be at a heightened risk for VTE who underwent CTPA based on a physician's judgment.

### Risk factors associated with PE

The present study results indicate that a history of VTE, immobility, thrombophilia, hormonal treatment or oral contraceptives, and obesity (body mass index (BMI) ≥ 30) are independent risk factors for predicting PE in gynecological inpatients. Surgery and active cancer are recognized as high-risk factors for VTE [38], contributing significantly to the scores in various thrombosis assessment models. However, these factors were not identified as autonomous risk factors for predicting PE in gynecological inpatients within the scope of the present study.

An analysis of the Registro Informatizado Enfermedad Tromboembolica(RIETE) and Medicare databases revealed that gender potentially has an influence on the risk factors associated with PE, as women afflicted with PE exhibited a higher propensity for immobility or hormonal treatment in contrast to their male counterparts. Conversely, the prevalence of cancer and cardiovascular diseases was observed to be greater among men [8]. This aligns with the findings of the present study. Other studies have also identified hormonal treatment as a risk factor for PE [34]. Moreover, existing research has confirmed the association between immobility and VTE [13, 31]. The present study revealed that a significant proportion of patients (87.0%) underwent surgery, which was associated with prolonged surgical duration, and a considerable percentage of patients (49.3%) had active cancer, both of which are known to potentially impede mobility and result in an extended period of bed rest. Additionally, Kandagatla et al. provided evidence demonstrating that a prior occurrence of VTE serves as an independent risk factor for PE [13].

Previous research suggests that an increase in BMI is linked to an increase in factors that predispose individuals to thrombosis and a decrease in the capacity for fibrinolysis [39]. A large prospective cohort study by Kabrhel et al. demonstrated a strong linear relationship between a BMI increase and the incidence of PE in women, and this association was not limited to individuals with severe obesity [40]. Furthermore, a meta-analysis by Jamal et al. proposed that obese patients have a higher risk of PE compared to individuals with a normal BMI (hazard ratio: 2.24, 95% CI: 1.93–2.60) [41].

Existing research on thrombophilia is currently limited. The PADUA model involves deficiencies in proteins C, S, and antithrombin III, as well as factors like Factor V Leiden(FVL) and prothrombin G20210A mutation. It is noteworthy that genetic susceptibility can potentially hold equal significance as environmental and clinical factors in terms of the risk factor for PE [42]. According to previous reports, deficiencies in proteins C and S have been linked to an increased risk of PE compared to that in the general population [43]. The present study findings support this idea, as 9.0% of patients were found to have hereditary thrombophilia. Multifactorial logistic analysis results indicated a strong association with PE, with an odds ratio of 50.34(95% CI: 15.05–168.38). Stefano et al. also discovered that patients with thrombophilia have a higher recurrence rate of PE [44]. Therefore, further investigation is needed to gain a better understanding of the relationship between thrombophilia and PE.

### K-M survival analysis

In the present study, both the PADUA and Caprini models predicted mortality events. However, when considering the three- and six-month follow-ups, the Caprini model showed a higher AUC for predicting mortality events compared to the PADUA model. Furthermore, the AUC in the Caprini model exceeded 0.7 at both the three- and six-month time points, indicating its potential utility in predicting prognosis for gynecological inpatients. Zhou et al. [31] also found that the Caprini model accurately predicts the mortality rate in hospitalized patients, yet there is currently little research pertaining to this subject matter. Prospective studies are needed to investigate the association between the Caprini model and mortality among gynecological inpatients.

## Limitations

Our study had several limitations. First, clinical data collection was conducted retrospectively, potentially introducing information bias. Second, the absence of data regarding the patients' receipt of anticoagulant treatment and occurrence of bleeding events hindered the ability to assess the effectiveness of the anticoagulant treatment in high-risk patients using the PADUA or Caprini scores. Finally, we conducted this retrospective study to identify a potential and valid model for the gynecological population, and more prospective studies are needed to re-validate the power of this model in the future.

## Conclusion

The present study discovered that both the PADUA and Caprini RAMs demonstrate efficacy in identifying high-risk individuals for PE among gynecological inpatients. However, from a comprehensive standpoint, it is advisable to prioritize the use of the PADUA model for the following reasons: 1) the PADUA model offers simplicity compared to the Caprini model, with easily obtainable information in clinical settings, and 2) it effectively predicts mortality rates. Nevertheless, the Caprini model outperforms the PADUA model in terms of mortality prediction. Additional prospective research is necessary in order to establish their stratification more precisely and minimize the utilization of unnecessary CTPA procedures.

### Supplementary Information

Supplementary Material 1.

## Data Availability

No datasets were generated or analysed during the current study.
