# Supplementary material for: Validation of a pulmonary embolism risk assessment model in gynecological inpatients: Clinical trial: A single-center, retrospective study
Source: Thromb J. 2024 Jun 5;22:47. doi: 10.1186/s12959-024-00616-5 (PMC11151723; doi:10.1186/s12959-024-00616-5)
Supplement: Supplementary file 1 — Supplementary Material 1. [file 12959_2024_616_MOESM1_ESM.pdf]

Supplemental Table1. IDDVT vs PDVT

	IDDVT	PDVT	P valve
PADUA score	5(2-6)	8(5-9.5)	0.002
Caprini score	6(4-7)	8(6-9.5)	0.000

IDDVT, isolated distal deep venous thrombosis; PDVT, proximal deep venous thrombosis

Supplemental Table2. Central PE vs Peripheral PE

	Central PE	Peripheral PE	P valve
PADUA score	3(2.5-5)	3(2-4)	0.049
Caprini score	7(6-9.5)	7(6-8)	0.271

PE, Pulmonary embolism;

Supplemental Table3. The association between the severity of PE and RAM

	PADUA	Caprini
PESI score	P=0.000 R=0.458	P=0.000 R=0.416
resence of symptoms	P=0.226 (0.89-1.61, OR=1.20)	P=0.207 (0.94-1.33, OR=1.12)
presence of hypoxemia	P=0.191 (0.92-1.56, OR=1.19)	P=0.505 (0.90-1.23, OR=1.06)
right heart load	P=0.208 (0.51-1.16, OR=0.77)	P=0.753(0.77-1.21, OR=0.96)
